# Using a person-centered approach in clinical care for patients with complex chronic conditions: Perspectives from healthcare professionals caring for Veterans with COPD in the U.S. Veterans Health Administration’s Whole Health System of Care

**DOI:** 10.1371/journal.pone.0286326

**Published:** 2023-06-23

**Authors:** Ekaterina Anderson, Renda Soylemez Wiener, Brianne Molloy-Paolillo, Megan McCullough, Bo Kim, J. Irene Harris, Seppo T. Rinne, A. Rani Elwy, Barbara G. Bokhour

**Affiliations:** 1 Center for Healthcare Organization and Implementation Research (CHOIR), VA Bedford Healthcare System, Bedford, Massachusetts, United States of America; 2 Department of Population and Quantitative Health Sciences, University of Massachusetts Chan Medical School, Worcester, Massachusetts, United States of America; 3 Center for Healthcare Organization and Implementation Research (CHOIR), VA Boston Healthcare System, Boston, Massachusetts, United States of America; 4 The Pulmonary Center and Department of Medicine, Chobanian and Avedisian School of Medicine, Boston University, Boston, Massachusetts, United States of America; 5 Department of Public Health, Zuckerberg School of Health Sciences, University of Massachusetts, Lowell, Massachusetts, United States of America; 6 Department of Psychiatry, Harvard Medical School, Boston, Massachusetts, United States of America; 7 VA Maine Healthcare System, Lewiston, Maine, United States of America; 8 Department of Psychiatry, University of Minnesota Medical School, Minneapolis, Minnesota, United States of America; 9 Division of Pulmonary and Critical Care Medicine, Chobanian and Avedisian School of Medicine, Boston University, Boston, Massachusetts, United States of America; 10 Department of Psychiatry and Human Behavior, Warren Alpert Medical School of Brown University, Providence, Rhode Island, United States of America; Monash University, AUSTRALIA

## Abstract

**Background:**

The largest nationally integrated health system in the United States, the Veterans Health Administration (VHA), has been undergoing a transformation toward a Whole Health (WH) System of Care. WH Clinical Care, a component of this system, includes holistically assessing the Veteran’s life context, identifying what really matters to the Veteran, collaboratively setting and monitoring personal health and well-being goals, and equipping the Veteran with access to conventional and complementary and integrative health resources. Implementation of WH Clinical Care has been challenging. Understanding healthcare professionals’ perspectives on the value of and barriers and facilitators to practicing WH Clinical Care holds relevance for not only VHA’s efforts but also other health systems, in the U.S. and internationally, that are engaged in person-centered care implementation.

**Objectives:**

We sought to understand perspectives of healthcare professionals at VHA on providing WH Clinical Care to Veterans with COPD, as a lens to understand the broader issue of WH Clinical Care for Veterans living with complex chronic conditions.

**Design:**

We interviewed 25 healthcare professionals across disciplines and services at a VA Medical Center in 2020–2021, including primary care providers, pulmonologists, palliative care providers, and chaplains. Interview transcripts were analyzed using qualitative content analysis.

**Key results:**

Each element of WH Clinical Care raised complex questions and/or concerns, including: (1) the appropriate depth/breadth of inquiry in person-centered assessment; (2) the rationale for elicitation of what really matters; (3) the feasibility and appropriate division of labor in personal health goal setting and planning; and (4) challenges related to referring Veterans to a broad spectrum of supportive services.

**Conclusions:**

Efforts to promote person-centered care must account for healthcare professionals’ existing comfort with its elements, advocate for a team-based approach, and continue to grapple with the conflicting structural conditions and organizational imperatives.

## Introduction

The Veterans Health Administration (VHA) is a subdivision of the United States Department of Veterans Affairs (VA) that serves eligible military Veterans and their family members. For over a decade, VHA, the largest nationally integrated healthcare system in the U.S., has been undergoing an unprecedented transformation to a Whole Health (WH) System of Care that promotes Veterans’ health and well-being in a person-centered manner. VHA’s WH system seeks to empower Veterans to collaborate with their care team as equal partners, encourage the use of self-care skills, and promote access to both conventional and complementary approaches to disease treatment and prevention [1,2]. The central element of the WH System implementation is WH Clinical Care–that is, primary and specialty care services offered in line with a WH approach, as opposed to the older disease-centered approach. WH Clinical Care encompasses four principles for approaching clinical encounters: (1) the state of the Veteran’s health and well-being is assessed in a comprehensive, whole-person way; (2) what matters most to the Veteran–their meaning or purpose in life–is elicited early and prioritized throughout the encounter; (3) the Veteran is supported in setting and pursuing realistic health and well-being goals that are related to what matters most to them; and (4) the Veteran is equipped with knowledge, information, and resources in support of these goals, which may include connecting the Veteran with other WH System of Care offerings–e.g., peer-facilitated groups or complementary and integrative health (CIH) providers [3].

VHA’s WH Clinical Care is fully aligned with the principles of person-centered care. We define person-centered care as an approach to care that considers each individual who comes into contact with the healthcare system as a person with a unique life context and aligns the care with the individual’s preferences, goals, and priorities [4–8]. In other words, patients are viewed as more than the diseases from which they suffer. Indeed, WH Clinical Care incorporates and scales up many of the key principles that the person-centered care movement has been advocating for many decades but that are not always explicitly captured in brief definitions–a holistic view of health and illness as affected by multiple factors; empowerment of the patient to engage in their own care (sometimes described as patient-directed care); and integration of non-medical and supportive services, to name a few [5,9–11]. In the United States, the urgency of attending to the health and well-being of the whole person–not just to diseases of specific organs or body systems–has recently been highlighted in a new strategic plan issued by the National Center for Complementary and Integrative Health (NCCIH) of the National Institutes of Health (NIH) [12]. Calls for making person-centered care a matter of U.S. policy have been issued for decades, including, notably, a recent proposal by the National Academies of Sciences, Engineering, and Medicine to scale VHA’s Whole Health model at the national level [7,13,14]. Similar developments are taking place globally [15–17]. In this context, it is more critical than ever that VHA’s experiences of building a WH System of Care are thoroughly described and analyzed, so as to allow policymakers, clinicians, and patient advocates in the U.S. and beyond to learn from VHA’s lessons. As a national-level, publicly funded health system, VHA’s experiences are relevant beyond the U.S. healthcare landscape.

Prior research shows that, despite the advances already made, implementing WH Clinical Care has been an enormous challenge for VHA, and the slowest component of the implementation so far [18,19]. Efforts to increase clinicians’ use of WH Clinical Care have had varying success [18,20,21], and many have argued that having a system that supports this approach to care is critical [22,23]. Much of the existing literature, however, has focused on the high-level organizational challenges of implementation as perceived by frontline clinicians and leaders [24,25]. It is essential to better understand frontline healthcare professionals’ perspectives on the meaning, rationale and practicality providing WH Clinical Care, because their conceptualizations have an immediate bearing on the success of implementation efforts.

While VHA envisions WH Clinical Care as applicable to all patient populations, it is a particularly natural fit for patients with complex chronic conditions. Such individuals face a range of functional and psychosocial challenges that WH Clinical Care is well-positioned to address, given its person-centered focus on supporting patients in pursuing a holistic vision of health and well-being in line with their values, goals, and preferences [26,27]. Chronic obstructive pulmonary disease (COPD) is a case in point. COPD is a complex, systemic condition that affects and is affected by all areas of the individual’s physical, psychological, and social well-being. Breathlessness, a common symptom of COPD, causes tremendous distress to patients and caregivers [28], and acute exacerbations of breathlessness may result in emergency room visits or costly hospital stays [29,30]. Individuals living with COPD also experience decreased everyday activity [31,32], social isolation [33–35], depression and anxiety [36–38], feelings of guilt and self-blame [39], and distress about the future [40–42]. A growing chorus of voices in the academic, clinician, and patient advocate communities has been calling for implementation of a person-centered approach wherein the clinical team empowers and supports the individual with COPD to pursue a better quality of life and a sustained sense of well-being, in line with this individual’s values, goals, and preferences [43–45]. Despite evidence in support of this patient-centered approach to COPD care [46–59] and its alignment with VHA’s WH Clinical Care model, such care is not widely practiced—either in the VHA or beyond. In this paper, we sought to understand how healthcare professionals across disciplines at VHA view the value of and the facilitators and barriers to providing WH Clinical Care to Veterans with COPD, as a lens to understand their receptivity to WH Clinical Care in general.

## Methods

We conducted a qualitative study to understand factors affecting the uptake of the Whole Health Clinical Care in outpatient care for Veterans with COPD. Between October 2020 and November 2021, we recruited staff members at a large urban VA Medical Center (VAMC) that includes both tertiary inpatient care and an extensive selection of outpatient services to participate in semi-structured interviews. We sought to interview healthcare workers across diverse services and disciplines that typically play an important role in managing outpatient care of Veterans with COPD, including primary care, pulmonary medicine, and palliative care. In alignment with the WH vision wherein whole-person care incorporates broader psychosocial well-being, we also interviewed staff in mental healthcare services (which are increasingly recognized as a key element of comprehensive care for patients with COPD), the chaplain service (as spiritual/existential concerns are well-documented in the population of interest), and the site’s Whole Health service, which is responsible for promoting and supporting the implementation of the Whole Health approach locally. As part of the larger study, we also interviewed Veterans with COPD. We chose not to integrate an analysis of their perspectives into the current manuscript as the emphases and range of topics covered in their interviews were so different as to merit a separate manuscript (currently in preparation). The study was approved by the VA Bedford Institutional Review Board (IRB), as well as by the IRB of the VA site where the data for this study were collected.

The interview guide (S1 Appendix) was developed iteratively with input from co-authors and drawing on the Consolidated Framework for Implementation Research (CFIR) [60] for guidance on the content and structure. CFIR was chosen for its comprehensive, multi-level conceptualization of implementation determinants, such as perceptions of the intervention (e.g., WH Clinical Care), the inner setting (e.g., specific VAMC and VHA as a whole), outer setting (e.g., the nation-wide push for patient-centered care), characteristics of individuals involved (e.g., healthcare professionals for the purposes of this study) and the process of implementation (e.g., engagement of VAMC in the WH System of Care implementation). Participants were invited to participate in the study via e-mail; e-mail addresses were obtained from internal VA address books and public VA provider directories. Interviews took place over the phone or Microsoft Teams, depending on the participants’ preference. Prior to each interview, the interviewer (first author) went over the study description and participants’ rights. Verbal informed consent was obtained prior to participation. All interviews were recorded with participants’ permission. After each interview, the interviewer took notes to capture the content and initial analytical reflections.

Interview data were analyzed using qualitative content analysis [61]. The first author coded the transcripts in the qualitative data analysis software ATLAS.ti using inductive codes (derived from the data). The inductive codes were then organized into larger categories corresponding to the four elements of WH Clinical Care described above. Throughout the coding and analysis process, the first author used memos to record insights about similarities and differences across the data set, as well as emerging concepts. Drawing on post-interview notes, memos, and coded data, the first author generated an initial set of themes, each of them pertinent to the four elements of WH Clinical Care. These initial themes were refined with the help of the senior author, who also reviewed the larger dataset to ensure that the themes adequately reflect the patterns in the data. The naming and content of the themes were finalized by incorporating several rounds of oral and written input of other authors.

## Results

We interviewed 25 individuals from a wide range of disciplines and services. The majority of participants were physicians (48%) (see Table 1 for more details).

**Table 1:** Participant characteristics (n = 25).

Characteristic	Frequency	%
***Profession***		
Physician (MD or DO)	12	48%
Nurse Practitioner (NP)	2	8%
Clinical Psychologist	5	20%
Clinical Social Worker	1	4%
Chaplain	3	12%
Other	2	8%
***Primary Work Setting***		
Primary Care	5	20%
Pulmonary	5	20%
Palliative Care	3	12%
Mental Health	3	12%
Whole Health	6	24%
Other	3	12%
***Years at VA***		
1–5 years	6	24%
6–10 years	8	32%
11–15 years	5	20%
16–20 years	2	8%
20+ years	3	12%
Not available	1	4%

Our overarching finding is that participants had lingering questions/concerns regarding each of the WH Clinical Care elements–namely, (1) whole-person approach to assessment; (2) eliciting what matters most; (3) supporting the Veteran in setting and pursuing personally meaningful and realistic health and well-being goals; and (4) equipping the Veteran with knowledge, information, and resources in support of these goals. The four themes identified in participants’ discussions of WH Clinical Care, each of them associated with the specific element of WH Clinical Care, are presented in Table 2, with their brief names intended to capture the question/tension in point.

**Table 2:** Summary of themes.

Theme name	Brief description of theme
Assessing the Veteran’s life context: How much is too much?	While assessing the Veteran’s life context was generally endorsed as valuable, participants differed with regards to the depth and breadth of inquiry, perceiving some areas/topics as problematic.
Understanding what really matters: To what end?	Participants varied in their perspectives on the value and purpose of inquiring about what really matters to the Veteran, with some treating this question as a first step to aligning the care with Veteran’s priorities and others using the information as leverage to motivate the Veteran to take up health-promoting behaviors. A few found the question altogether irrelevant or otherwise hard to use.
Goal setting and personal health planning: Whose job is it, anyway?	Although participants were open to and, in some cases, experienced with setting shared goals with the Veteran, they had concerns about the more comprehensive and laborious personal health planning process.
Equipping Veterans with resources: How to navigate all these options?	Participants were generally open to the idea of connecting Veterans with COPD to services that support their well-being holistically, yet they reported knowledge gaps and raised concerns about both the relevance and logistics of making such connections.

The themes are presented below with illustrative quotes; participant IDs are presented in the service-discipline-number format (e.g., PULM-PHYS-01 referring to the first physician in the pulmonary service interviewed over the course of the study).

### 1. Assessing the Veteran’s life context: How much is too much?

When asked to describe their typical appointments with Veterans who have COPD, many participants described their efforts to understand the Veteran’s life context, especially during the first appointment–a practice that broadly aligns with the WH Clinical Care model. Incorporation of life context in clinical conversations, however, varied in scope and focus. In some cases, the details of the descriptions fully embody WH principles. For example, a primary care provider who was very familiar and comfortable with the WH terminology described a comprehensive approach wherein the full scope of the Veteran’s social support needs is explored, beyond the narrow focus on the disease:

“…I use a Whole Health template to take kind of like a more extensive social history than I might otherwise. And what I like about it, is that… it’s, like, really a fuller account of human flourishing and so, you know, asking about things like what’s most important to you in life and social stressors and social support. <…> I wanna know where they live, of course. I wanna make sure they have reliable transportation. <…> …sometimes, you know, there are a lot of veterans who aren’t working or maybe… who are medically retired because of service related-injuries or whatnot, and… there’s a lot of support in terms of like… supportive work therapy or whatever” (PRIMARY-PHYS-04).

This quote stands in contrast with one from a different clinician, a pulmonologist who was both sympathetic toward WH and self-admittedly not very knowledgeable about it:

“…I don’t necessarily use the terminology of Whole Health care, but in general, my style in trying to take care of patients is to try to connect with them on a personal level and… <find> out what’s going on in their lives, and often that… may influence what’s going on with their respiratory symptoms and their COPD. The issue of smoking cessation is completely wrapped up in their lifestyle and… what stressors they’re encountering. So… it’s pretty routine for me to ask about their environment in terms of… what could be affecting their breathing. So, the presence of animals in the house, dust, mold, the condition of the house, etc. You know, smokers in the house whether it’s the patient themselves or family members so I get a… rough idea what’s going on <in> the house or… wherever they’re living or if they’re unstable in their housing situation” (PULM-PHYS-01).

This interviewee, like the previous one, recounted a comprehensive assessment of the Veteran’s life context, which is broadly aligned with WH principles, yet they placed emphases in a notably different way. Specifically, all of the questions described concern factors in the Veteran’s environment that may affect their respiratory symptoms. Such assessment would result in a more circumscribed picture than the “fuller account of human flourishing” described by the participant quoted earlier.

A related, yet distinct point of tension came up in the context of assessing for mental health and socioeconomic difficulties. A primary care provider expressed discomfort with the idea of probing into this area, perceiving it as difficult to address within a physician’s scope of practice:

“…some questions you don’t want to ask because you can’t address them. You know, it is very difficult for me as an MD physician. If… something is wrong with their housing… all I do really is, ‘oh, let me have you talk to a social worker.’ You know… sometimes you don’t want to ask the question if you don’t want to know the answer, or at least know how to answer the question for them or how to actually help them” (PRIMARY-PHYS-02).

Several participants reported a similar uneasiness around inquiring about spiritual or existential concerns that are sometimes present in patients with COPD, such as shame, guilt, and fear of death, during routine appointments. For example, in response to the interviewer question about whether such topics come up in the appointment, a primary care provider answered:

“Yeah, but we really don’t have enough time for that. …this would require a separate visit, you know, because you open up a door that you have to close it very fast and that would be not appropriate to do. Obviously, if somebody’s coming with some concerns or symptoms, and we can use large part of the visit for discussion for that particular aspect of their life, that’s a different thing but not during the routine visits. So… we usually don’t go–if there is a sense that there is a need for that, we have enabled, you know, help from our Behavioral Health” (PRIMARY-PHYS-01).

A notable counterpoint to the routine avoidance of this sphere of life/well-being came from an interview with a WH Coach (a WH service staff member whose role involves delivering health and well-being coaching to Veterans), who modeled a thoughtful approach to framing the conversation about spiritual concerns:

“…what’s the best practice for me… is to kinda initiate that conversation by explaining to them that for some this might be about your religion, but for others it’s about a connection… to something outside of yourself. <…> So… for Veterans who are not religious, it doesn’t have to feel awkward to them because it’s not just about religion. You know, it’s really about your soul, it’s about connecting. And once you…have that conversation, I think it’s much better received than, you know, to just come in and say, ‘We’re going to talk about spirit and soul,’ and the first thing you do is roll out the Chaplain. <…> So, although we have Chaplains available at the VA to kinda come in and speak to Spirit and Soul, you know, I think anyone can do it because, again, it’s really that connection that the Veterans resonate with and connect with” (WH-04).

In sum, while there was a buy-in into the need to understand the Veteran’s context, there were disagreements as to which elements of this context are deemed as relevant and appropriate to assess.

### 2. Understanding what really matters: To what end?

A major element of WH Clinical Care–and person-centered care, more broadly–is the emphasis on understanding “what really matters” to the patient. In this paradigm, “what really matters” refers to the deepest motivating force running through the Veteran’s life, the Veteran’s innermost goals and priorities. Inquiring about whether participants ask about what really matters to Veterans as part of their practice has produced a variety of responses–from descriptions that would not be out of place in WH training materials to accounts that expressed unease or confusion about this idea. Specifically, we identified three types of perspectives that are described in detail below:

1. Using the “what really matters” question to take the Veteran’s lead
2. Using the question to gain leverage in the service of the clinician’s agenda
3. Challenge in effectively using the question

### 2.1. Taking the Veteran’s lead

On one end of the spectrum are participants who appear to have fully internalized the imperative to elicit the Veteran’s life priorities–they ask what really matters in order to take the Veteran’s lead. For example, a pulmonologist involved in the pulmonary rehabilitation program described how the program staff seek to understand what the desired level of everyday functioning would be for the Veteran, in order to help each Veteran realize their unique personal vision:

“…yeah, we <ask> them, ‘what’s important to you?’–you know… ‘what <does> a good quality of life mean to you’ and I think <if they say> ‘I’m totally happy sitting on the couch. I just don’t wanna be short of breath when I’m sitting on the couch.’ Then, okay, that’s very different from someone who tells me… ‘I used to being able to… go on walks with my grandchildren and I can’t go out anymore.’ …So, yeah, it’s… what do they value in their lives, what do they want you to do, and is there a way that we can help them achieve that” (PR-PHYS-01).

This passage is exemplary as it expresses an understanding that what may be important to one Veteran is not important to another–an issue that is at the heart of the debate around the extent to which the patient’s goals and preferences should be prioritized by the clinician. A similar approach was expressed in all of our interviews with palliative care providers, who saw their role as supporting Veterans in doing what really matters to them:

“…thinking about the veterans I’ve cared for… over the last year with COPD, many of them… are just so advanced by the time they get to us that they’re not able to get out garden and drive and do all the things that *used* to matter most. So, then it’s what matters most *now*. And a lot of it is spending time with family. …like, this… Veteran who was in our unit just temporarily, and his wife was having this prolonged admission, he… really, really wanted to go home. <…> …what mattered most to him was being back in his home environment… so we discharged him home. So, you know, I think as people progress what I found is the things that are—matter most to them are very—more practical, more tangible. <….> So, the <what> matters most can be more kind of vague and sometimes it’s very concrete and so… even taking care of these practical things goes a long way” (PALL-PHYS-03).

### 2.2. Leveraging what really matters

A contrasting approach involved strategically using the information about what matters to the Veteran in service of the clinician’s agenda. For example, one primary care provider recounted routinely using WH “techniques” to encourage lifestyle change in Veterans with chronic pain and diabetes, but *not* those with COPD. In the quote below, this clinician shares that their decision to participate in the interview was motivated by the desire to learn more about using a WH approach with Veterans with COPD:

“I… use Whole Health a lot… and I tend to use it more, like, in pain management; I <also> used it a lot <in> lifestyle change around, like, diabetes, of getting involved in exercise and working on your diet and kind of having a Whole Health coach meet up with you periodically <…> …it was one reason I… was interested in talking to you is ‘cause it is not one that I had thought about as far as, like, inhaler adherence or even… getting beyond just smoking cessation…” (PRIMARY-NP-01).

This participant is clearly familiar with and enthusiastic about WH language and tools. At the same time, when this interviewee contemplates the prospect of asking Veterans with COPD about what matters most to them, they see it, in a somewhat narrow manner, as a tool to promote inhaler adherence and smoking cessation, rather than as an invitation for an open-ended exploration of the Veterans’ own priorities which might or might not fully align with these best practices. This interviewee’s approach thus displays a second understanding of the rationale for the “what really matters” question–to leverage and support Veteran’s own motivation to make healthy behavioral changes. In other words, the clinician asks about what matters most to the Veteran in order to facilitate Veteran’s engagement in the goals that the clinician has already designated as important (“lifestyle change”).

This understanding was also present in the words of another participant, a psychologist in mental health care services, who was knowledgeable about WH yet also described using the question about what is important as a way to promote treatment adherence, thus subordinating “what really matters” to a biomedical rationale:

“I think it’s a great question because what it’s really speaking to is… what’s important to the patient, and what’s important to the patient is going to drive their health behaviors and their choices. They may not recognize that, but that’s where your leverage is. If they say what’s important to me is, I don’t know, walking with my grandchildren or… something like that, then you’re going to be able to help with their motivation and their treatment adherence through this value of what’s important to them” (MH-PSYCH-01).

### 2.3. Challenge in effectively using the question

Finally, some participants found it difficult to effectively inquire about what really matters to the Veteran during a clinical encounter. This concern was most fully demonstrated in an interview with a primary care provider that is worth quoting at length. First, the interviewee explicitly takes issue with the narrative of WH trainings and reports abandoning the question of “what really matters” after receiving excessively vague answers that were difficult to relate to the rest of the clinical conversation:

“…it never works the way that it has been portrayed to work in the training, and I actually find the Veterans are… as… confused by the question as we are, you know… they will always say kind of my health, my family and you get much beyond that… and then the connection to how do I connect that to your COPD is just not there. I try to force them to have the connection there anyway in a way that doesn’t feel natural and doesn’t help the conversation… Or it’s just hard for me to connect COPD to that thing that maybe they there are not able to do as well” (PRIMARY-PHYS-02).

The same participant then endorsed the more scripted/concrete elements of WH Clinical Care:

“<I> actually like the more action-oriented parts of Whole Health … like, ‘okay, great, it sounds like Tai Chi might be good for you,’ and I’m not gonna try to make an artificial connection to why. <…> …there’s the poster that we put up that has the different circles that show all those different components and I have been surprised. There are Veterans that will see that and say, ‘oh yeah, that’s a different way of thinking about it.’ And I haven’t engaged with that framework, those more kind of domain-specific questions as much as might be recommended.…there is value I can see in putting more meat on the bone of that kind of single question, what is most important to you. When you break it down into the different circles of health that might help them, that might help that conversation go better and to be honest it is not a part of my routine practice. <…> …maybe I just need to be shown the light, shown some examples where it really, really made the difference and I just haven’t had that experience yet” (PRIMARY-PHYS-02).

The difficulty with making the question about “what really matters” work was acknowledged by other participants. Two primary reasons for this challenge were offered. One was that the question was poorly contextualized; i.e., little guidance was provided for how to incorporate it into a routine appointment.

“I think we need to learn how it can be useful as a tool. I don’t think it’s valuable if it’s just a question we ask everybody for the sake of asking the question because honestly when you ask most patients what’s important to you they’re gonna say their family. …so I—we just need to know, okay, glad that I asked you that, but, like, what do I do with that information? How is that helpful to me?” (PRIMARY-PHYS-03).

“I think what maybe gets lost is, like, what do you do with that information and… what does it mean. <…> I mean, I think it’s a weird question. …I love it *and* I think it’s a weird question to ask because it has no context around it, right? …I’m not surprised at all that providers are, like, ‘what do I do with this?’ <…> …could it be part of… a psychosocial needs assessment that isn’t just… plopped in the middle of an intake about other things, …in a more dedicated… 10- or 15-minute conversation that is more focused on psychosocial stuff? And, like, you could put this ‘what matters most question’ in that… and you need to give providers… options of what to do next that isn’t too burdensome, and, like, what do you do with that information?” (MH-PSYCH-01).

Another explanation, interestingly, pointed to the challenges of asking Veterans who may not be prone to reflection to share something as complex and intensely personal as what matters most to them:

“…a lot of Veterans don’t know the answer to the question which is surprising and interesting because those are some of the people who end up in my office ‘cause… they’ve lost their sense of what’s important or they don’t feel like they can… live a life that is important anymore. <…> So, I think it may … just not be registering with in their lives. Like, who, like, they may have never been one of those people who stepped back and, like, explored this bigger question, especially male older adults. Like, I think… a lot of them are not super-emotional-minded or… even that insightful… …they don’t know what to do with those questions. But some of them do, it just depends” (MH-PSYCH-01).

### 3. Goal setting and personal health planning: Whose job is it, anyway?

In the WH Clinical Care model, the Veteran and their provider or clinical team are encouraged to collaborate on setting shared goals based on what really matters to the Veteran. These goals are then, ideally, documented in the Veteran’s chart to facilitate information sharing across clinical team members, as well as to enable goal follow-up and adjustment. Although participants were open to and in some cases experienced with setting shared goals with Veterans, several raised concerns about feasibility, and there were differing perspectives on the appropriate scope of and distribution of responsibility for goal setting and health planning.

Several interviewees described how they deliberate with the Veteran on the best course of action in light of the Veteran’s priorities and preferences as a routine part of their practice, although they did not always use WH-specific language such as SMART goals. For example, a pulmonologist specializing in sleep issues shared the following:

“…often, I’ll have patients who say, well, ‘I really want to lose some weight.’ <…> And… so we’ll go that route instead of positive airway pressure. And I say, you know, ‘let’s reassess in a few months and see who you’re doing with that.’ There are other patients who really want to choose the CPAP, and even though they fail at it for months and even years sometimes, they don’t want to consider a different… treatment option. …And sometimes, they just <do not>… want treatment at all.” (PULM-PHYS-02).

Although the concept of setting goals was relatively familiar, the interviewees were more concerned about the feasibility of conducting personal health *planning* as a comprehensive and longitudinal process. For example, a pulmonologist said the following in response to a question whether they had seen or added to a Veteran’s personal health plan:

“No. [Laughing] I have not. It’s possible that I have seen it… and I was not–to be honest–you know, motivated to go look at it because we spend so much time in front of the computer that I try to do what I need to do to get the computer work done. So, I have not put together a… personal health plan for a patient. That would be, you know… an example of a perception of a specialist deferring that to the primary care physician because I think it’s wrapped up in their… management of their hypertension, management of their diet, their hyperlipidemia, their diabetes and so forth beyond just my recommendation that they walk or that they exercise that they, you know, whatever I can do to enroll them in either MOVE! Program or <pulmonary> rehab” (PULM-PHYS-01).

In other words, while this participant was open to the idea of making a few recommendations or placing referrals, they saw health planning as more appropriate for a primary care provider to take on. Another pulmonologist expressed a similar opinion, albeit in a more forceful fashion. What is notable about the passage below is that this participant sees personal health planning as misaligned with their role in the context of regular outpatient care for Veterans with COPD yet endorses it as their dominant approach in the context of severe, end-of-life COPD:

“It’s ridiculous. <…> I’m sorry, I’m being completely honest. …I’m a specialist so I am not the primary care doctor… …when my patient is coming to see me, they… want me to address their lung issues, their COPD issues… <….> Let me amend it this way. So, there are patients that I see in my clinic for COPD that are maybe severe end-stage who are at the tail end of their life where… I’m managing their health status in general. <…> …for these patients… my framework shifts, okay? I’m still the specialist but, yeah, absolutely, in that situation the framework of understanding, you know, what is more important to the patient, what are their goal <so> that framework applies, sure” (PULM-PHYS-03).

This notion of personal health planning as lying outside of the pulmonologist’s regular role with regards to providing care to Veterans with COPD was challenged by a primary care provider:

“I think that if you’re an expert what an expert does is understand all angles of a problem and be able to comment on it all, and use it all and integrate it all… I think it’s easy for people to say no, not me, no, not my specialty, but… who else’s specialty should it be, if not the expert’s on all of the things that contribute to COPD and all of the things that are available for COPD treatment?” (PRIMARY-PHYS-03).

The issue of appropriate division of labor with regards to personal health planning came up in the interviewees in the WH service, as well. Several interviewees whose role involved clinician education felt that it would be appropriate for a provider to set a few shared goals during the appointment yet rely on the WH service to follow up with the Veteran on goal progress:

“I think the peers and the coaches are the answer to providers who say, ‘I don’t have time for this.’ You know, the providers can introduce whole health and then can send veterans toward…the coaches to do much more in-depth work over time around Whole Health… so… a coach <can> meet with a veteran one-on-one to continue to talk about goals and values and what’s important. <…> So, I see… the coaches as absolutely being able to kind of expand the reach of Whole Health because they do have the time. That’s their job description. So, taking some of the burden off the providers who don’t have the time, right, to talk every week about Whole Health to individual patients” (WH-06).

Offering a counterpoint, a WH Coach we interviewed spoke to the importance of a personal relationship with the clinician–in other words, instead of the one-directional approach (the coach takes over where the clinician leaves off), the coach and the clinician engage in a give and take dynamic, deliberating together over how to empower the Veteran:

“I meet with different providers all the time because they call me and say, “Hey… I have this situation.’ You know, ‘I’m trying this with the guy.’ You know, ‘I’d like you to give it a shot or have a conversation with him.’ And oftentimes… because of the relationships that we have, you know, this Veteran’s getting that wrap-around support, not only from the Health Coach, but that provider’s buying into it, and that provider’s also, you know, starting to use some of the language and changing the conversation, as they say, around healthcare” (WH-04).

### 4. Equipping Veterans with resources: How to navigate all these options?

Most interviewees felt that discussing or providing Veterans with COPD with referrals to a broad range of services that may support their well-being was worthwhile, yet some challenges were also raised. To obtain a comprehensive picture, we inquired about services that are usually seen as falling under the WH umbrella, such as complementary and integrative health (CIH) and WH coaches, but also other services, including palliative care, mental health care, and chaplaincy. Two types of barriers to referrals came up in the interviews: (1) barriers related to the interviewees’ knowledge and attitudes about service referrals and (2) barriers related to logistics. For this manuscript, we only focus on the former as the latter are highly granular and specific to VHA and the specific VAMC setting.

Generally, participants were open to the idea of connecting Veterans with COPD to services that support their well-being holistically. In a few cases, however, interviewees said that they had not previously considered the possibility and perhaps lacked the knowledge of how these services would help patients with COPD, but would now be interested in doing so in future:

“You know, I honestly haven’t recommended it specifically for Veterans with COPD, but I would imagine for our… less severe cases…. it could be really helpful as far as maybe like some pneumonia prevention and getting some movement, getting up and moving around a little bit and helping their immune system with some blood flow ‘cause they aren’t really vigorous exercises, especially Tai Chi… <…> So, I’m sure that more severe COPD is probably—that’s inappropriate, too late, but for our earlier diagnoses, more well-controlled, where definitely exercise is still important, I think that, you know, it’d be interesting to see if it could help prevent pneumonia” (PRIMARY-NP-01).

For example, none of the participants interviewed were aware of or previously considered connecting Veterans with COPD with a chaplain outside of the inpatient and/or end-of-life context:

“I’ve never thought to bring it up in the outpatient setting… …I do attend on the inpatient medical wards and I know, sometimes I’m kind of struck by, ‘oh, the chaplain came by and saw the patient today and left a note,’ and it just kind of it stands out as something like, it’s just like a totally different world than what I practice. So, to be honest I’ve never considered bringing that into outpatient care. And, you know, I don’t think a Veteran has ever asked me to and I refuse, I don’t think I would ever do that, it’s just never occurred to me and it’s never occurred to me to suggest it if I detect that there was maybe spiritual need or maybe even spiritual distress that was in play” (PRIMARY-PHYS-02).

One of the chaplains interviewed attributed this phenomenon to the inadequate awareness of what spiritual care is or how it is relevant to the Veteran’s team’s ability to provide whole-person care at all stages of life:

“There is huge poverty of understanding. And a lot of denialism that happens. People know what they know, and they are less inclined to learn what they don’t know. You know, people will trivialize it with spiritual care because they don’t understand it. Maybe because they… figure for themselves, ‘if I don’t have any use for it, you don’t have any use for it.’ But……the least you can do when you don’t understand is to learn. <…> …because our target is the same. …we’re here to serve one individual and that’s the Veteran. If we understand that this Veteran has different dimensions and have been in several different places, then we cannot just compartmentalize ’em and say, ‘oh no, I’m just a psychiatrist.’ <…> So part of my goal is… that we can communicate… much more fluently… and we can tackle it from… different… specialty areas but knowing that… we are here to serve one individual” (CHAPL-01).

Some participants also felt that Veterans with COPD themselves may see well-being offerings like CIH as unrealistic to pursue given the severity of their condition and/or logistical barriers, despite the obvious relevance of these offerings for improving their quality of life:

“A lot of them have very little breath. <….> At rest, they’re just totally fine, but they’re sick and tired of sitting still. They don’t want to be at rest anymore. They remember when they could walk three miles a day, and they want to do that. They remember when they could wash the dishes without becoming winded. They remember when they could prepare dinner. They can’t stand to look out the window and see their wife shoveling the leaves because they want to do it. This is the pain of COPD. <…> But… they don’t like to leave the house, some of them. <…> Some of them have a car, some of them don’t. Some of them need to take their oxygen with them everywhere they go, and they’re afraid the tank will run out, you know. So, there’re a lot of barriers… and a lot of them are old, and they’re going to say, ‘Ach, what do I need with meditation?’ Or, like, ‘Yoga, that’s for young people.’ You know, I haven’t done a good job of selling it. I think that most of them *could* benefit, is the truth” (PALL-PHYS-01).

In such scenarios, conveying the benefits and relevance of such services requires knowledge and tact on the part of the provider. One of the WH Coaches interviewed gave an example of displaying such tact:

“So, I kind of explain what it’s like to be in the class. …I’ll make some recommendations of, ‘You have COPD. Avoid the hot Yoga; that is not going to be something that is in your wheelhouse to do right now.’ And then I’ll explain what it is and the benefits to them, as well, of, you know, typically people with COPD are older. Typically, they have some form of mobility issue. So, explaining that, you know, ‘This is going to help stretch you out a little bit. It’s going to work on strengthening some of your core. They do a lot of breathing during it, which can, of course, have a positive impact on your overall breathing.’ With the Tai Chi, I go into explaining, you know, ‘It has a lot of help with balance,’ ‘cause, again, something I’ve noticed is a lot of patients with COPD are also having some form of unrelated balance issues. So, again, explaining, ‘You know, this can help your balance. It can help your posture, which if you’re standing up straighter, your lungs are going to be a little bit bigger; might help you with some of the breathing’” (WH-02).

A final barrier to placing referrals to services for holistically supporting Veterans’ well-being that was commonly mentioned was lack of awareness that these offerings are available:

“So, I haven’t really thought about it… although I’m a yogi myself… …I just don’t necessarily think about referring…. I don’t have a problem with it… it never really occurred to me because I think I just wasn’t aware of it. So, I think that I probably am not aware of most of the offerings that would be available” (PULM-PHYS-01).

## Discussion

In this paper, we explored the multidisciplinary perspectives of VHA healthcare professionals on using WH Clinical Care with Veterans with COPD as a lens for investigating their attitudes and experiences with WH Clinical Care for patients who have complex chronic conditions, more broadly. We found that our interviewees experienced tensions and uncertainties across all four elements of WH Clinical Care: from person-centered assessment to understanding what really matters to Veterans, collaborative goal setting, and equipping Veterans with resources to holistically support their health and wellbeing. These challenges reflect a complex interplay of constructs across all domains of our initial analytical framework, CFIR–from the inherent characteristics of WH Clinical Care as an intervention (its complexity and adaptability) to the features of both the inner setting (especially the quality of professional networks and communication, but also organizational culture and implementation climate) and the outer setting (particularly patient needs as understood by our interviewees), alike. Individual characteristics of our participants, and especially their knowledge and beliefs about WH, also undoubtedly shaped their perspectives.

Our work carries several important implications that are relevant not only to VHA’s ongoing efforts to infuse a WH approach throughout its system of care, but also to other organizations and systems that are concerned with implementing person-centered models of care–both with regards to patients with complex chronic conditions like COPD and on the system level, as a whole. We elaborate on these general lessons below, focusing on three areas in particular: (1) the paradox of selective implementation; (2) the uncertainties around division of labor and coordination; and (3) the challenge of navigating the inherent tensions between the ethos of person-centered care, on one hand, and the logics of biomedical rationality and economic expediency, on the other.

Our first finding of interest is that our participants felt affinity with some elements of WH Clinical Care but not others (for example, a physician may be open to placing a referral to a CIH provider but *not* to engaging in personal health planning around what really matters to the patient). In other words, instead of perceiving WH Clinical Care as a single paradigm to be followed in its entirety, healthcare professionals may draw piecemeal on specific tools and concepts. This selective approach to WH Clinical Care is not necessarily at odds with VHA’s own vision. The WH Clinical Care implementation efforts to date have been built on the premise that many clinicians may already be utilizing at least *some* person-centered care principles in their practice, even if unwittingly or inconsistently so. Indeed, the WH Implementation Guide reads, “Often you may discover that a person or team’s practice *already includes elements of Whole Health Clinical Care*. Meet clinicians where they are, connect Whole Health with the work they are already doing or are required to do, and partner with them to establish goals to help them move towards a *fully transformed* Whole Health Clinical Care approach” [3] (emphasis ours).

The phenomenon of selective uptake of WH Clinical Care can be viewed through two contrasting, yet complementary lenses. On one hand, it presents an opportunity: Not treating WH Clinical Care–or person-centered care, more broadly–as an all-or-nothing proposition may empower clinicians to incorporate those elements of the approach that are most aligned with their established interests and strengths into practice. Paradoxically, however, this selective uptake of person-centered care principles and practices may create a challenge of its own. In implementation science terms, it is the challenge of balancing adaptability vs. fidelity: successful implementation initiatives must tailor an intervention to fit the context while *also* preserving the intervention’s core, indispensable characteristics, yet the boundary between the core and the periphery may be far from obvious [60,62,63]. In the case of complex, multi-level interventions such as WH Clinical Care or any other person-centered care initiative, this ambiguity is all the more pronounced. The biomedical paradigm is strongly entrenched in healthcare settings. Therefore, there is a risk that clinicians may end up using person-centered care practices sporadically and without fully embracing the spirit of person-centered care, which would defeat the very purpose of implementation. Any educational and outreach efforts undertaken to support the implementation of WH Clinical Care–and person-centered care, more broadly–must not only support healthcare professionals in incorporating select person-centered practices and tools into their established approach, but also encourage them to critically rethink the approach *itself* so as to promote a true paradigm shift from the biomedical model to a person-centered one. i Individualized coaching or audit and feedback interventions [64,65], used iteratively and over a period of time, may be well-suited for this purpose, but larger culture change interventions are also crucial.

Our second finding with broader implications concerns the uncertainty about an appropriate division of labor between various types of healthcare professionals in providing WH Clinical Care. Some of our participants seemed to think that the responsibility for the entirety of WH Clinical Care lies on the physician’s–particularly, primary care physician’s–shoulders, which was, understandably, perceived as burdensome. Others perceived WH as mainly the responsibility of other individuals–PACT nurses, WH Coaches, other WH service employees (e.g., CIH instructors, WH educators)–and reported little to no integration of a WH approach into their practice. This finding speaks to the importance of a coordinated approach across multiple disciplines and services in implementing WH Clinical Care. Using the language of normalization process theory [66], an approach that seeks to describe how interventions get embedded into the everyday fabric of practices within an organization, WH implementation efforts are yet to achieve a sufficient degree of either relational integration (seamless incorporation of the innovation–in this case, WH–into the existing networks of professional relationships between individuals and groups) or contextual integration (integration of the innovation into the existing organizational context, including structures and practices).

This challenge is hardly unique to the VHA setting. Many systems within and outside the U.S. are grappling with the imperative of scaling up person-centered care principles beyond the patient-clinician dyadic encounter–i.e., making care coordination itself person-centered [67–70]. In VHA and beyond, overcoming this challenge would require an intentional effort to leverage teams and collaborative relationships more broadly within and across clinical settings. Approaches such as relationship-centered care [71] and relational coordination may be well-poised to help guide or at least inform such efforts. In general, however, while care coordination and/or integration are enshrined in some patient-centered and person-centered care frameworks, there is yet insufficient guidance around the best practices for coordinating care in a way that is consistent with what really matters to the patient, and there are still numerous opportunities for greater conceptual clarity that can be addressed by future research [72].

Finally, our work highlights both the importance and challenges of explicitly attending to rhetoric and discourse of person-centered care in general and the WH model in particular [73]. Specifically, our findings highlight an important tension at the heart of the WH initiative. In our interviews, we uncovered two perspectives. One embraces the ethical imperative of structuring care around patients’ values, goals, and needs, whatever these may be. The other, however, sees WH Clinical Care as valuable *only to the extent that it helps achieve a predetermined objective*–Veterans’ compliance with treatment and engagement in healthy behaviors, more broadly. These contrasting views, we posit, derive from the lingering tension in the discursive framing that VA’s Office of Patient-Centered Care & Cultural Transformation (OPCC&CT) has adopted in its advocacy for WH–a tension that can be seen in broader discussions around person-centered care beyond the VHA context. WH promotional and educational materials tend to emphasize that the use of WH Clinical Care by healthcare providers would *both* create a meaningful, supportive environment for the Veteran *and* result in downstream outcomes that are desirable from the healthcare system standpoint and underpin healthcare providers’ performance metrics (i.e., improvement in clinically meaningful outcomes, reduced care utilization, lower cost burden).

It is understandable that the case for person-centered care may need to be supported with arguments that are compelling from a biomedical and economic standpoint. However, we argue that efforts to implement person-centered care–in the VHA, as well as beyond–can benefit from a more attentive and intentional approach to discourse and framing. Research has shown that patient-centered and person-centered care language can be coopted and leveraged in the service of a disease-centered and provider-centric agenda [74]. Outreach and education efforts ought to spend more time unpacking the tension between the biomedical and the person-centered models. For example, training programs could incorporate working through and debriefing on s scenarios wherein the ethos of person-centered care *clashes* with the logics of biomedical expediency and economic rationality instead of almost exclusively dwelling on situations when the two are aligned. Vignettes and case studies could explore how to navigate situations when the patient’s values, goals, and priorities turn out to not fully align with the clinician’s vision for what may be best for the patient. The training could then emphasize that a truly person-centered approach requires that the clinician and patient build on a foundation of trust and explore how clinical expertise can be leveraged *in the service of* the patient’s goals. Such vignettes could even show how the areas of misalignment or disagreement may shrink over time, as long as this possibility is not overstated in excessively idealistic terms.

On a more fundamental level, however, such educational efforts would not resolve the underlying conflict between the overt endorsement of person-centered care and the organizational priorities and incentives that may be directly at odds with person-centered care principles.. It appears that institutional person-centered care implementation efforts are plagued by a contradiction: they are targeting institutions whose entrenched assumptions and routines reflect a biomedical (disease-centered) paradigm, a directive rather than partnership-oriented approach to the patient-clinician relationship, and a preoccupation with minimizing costs and maximizing efficiency above all else. The tension between the ethos of person-centered care and organizational structures and priorities is, once again, not unique to the VHA setting, let alone to person-centered care for patients with COPD, and has been noted in such diverse contexts as home-based care for older adults, care for individuals with kidney failure, diabetes self-management programs, and others [75–78]. When structures and norms that are at odds with person-centered care are still in place and unquestioned, implementation efforts may succeed in transforming healthcare workers’ beliefs about the value of being person-centered and even some of their practices, yet the gulf “between *knowing* and *doing* person-centeredness,” as Franklin and colleagues put it [79], would remain. No simple solutions here can be offered beyond making a larger observation that as VHA and other systems in the United States and beyond progress in their person-centered care implementation efforts, these tensions may become even more overt and profound, stimulating difficult yet important conversations.

The main limitation of our study is that we collected our data at a single–albeit large and influential–health system in the United States, the Veterans Health Administration. The VHA possesses organizational characteristics that make it stand out in the U.S. healthcare landscape (e.g., national-level integration, focus on military Veterans and their families/caregivers, concern with providing comprehensive care with robust mental health and social support components). Additionally, the VHA is embedded in the U.S. sociocultural and political-economic context, which differs in significant ways from healthcare organizations and health systems in other regions of the world. However, we argue that the overall implications of our study are broadly relevant and transcend both the VHA and U.S. context. The importance of going beyond the clinician-patient dyad to leverage team-based and inter-service care coordination has been broadly recognized and incorporated into numerous existing conceptual frameworks of person-centered and patient-centered care [6,9,80]. The difficulty of integrating person-centered/holistic approaches into largely biomedical/disease-centered systems has also been described in various settings worldwide [4], and may indeed be one of the foundational challenges at the heart of person-centered care implementation. Finally, the tension between the ethos of person-centered care and the logics of economic efficiency is hardly a VHA- or U.S.-specific phenomenon. Indeed, health systems in other parts of the world, including Europe and the U.K., have been under an increasing pressure from austerity policies [81,82].

The other key limitation of the study is that we sought to explore healthcare professionals’ perspectives on the use of WH Clinical Care with patients living with complex chronic conditions through the lens of a specific condition–COPD. This focus has enabled us to obtain rich, example-specific perspectives. At the same time, however, we were not able to explore all aspects of WH Clinical Care for patients with complex chronic conditions due to the enormity of the topic. While we maintain that the key insights shared by our interviewees are broad and not COPD-specific, it is possible that we would have heard about different challenges and different understandings of WH Clinical Care, had we chosen to select a different condition or several conditions as the lens for our inquiry.

## Conclusion

We conducted a qualitative research study to understand the perspectives of healthcare professionals in the largest nationally integrated health system in the U.S., the Veterans Health Administration, on practicing Whole Health Clinical Care with Veterans with complex chronic conditions, using their experiences of providing care to Veterans with COPD in particular as the guiding lens for our inquiry. We identified questions and concerns that participants had regarding each of the four components of WH Clinical Care. We further explored the broader relevance of our findings, including the implications of the phenomenon of selective uptake of person-centered care elements by healthcare professionals for education and outreach efforts, the importance for a team-based approach for sustainable person-centered care implementation, and the imperative of grappling with the lingering tension between the ethos of person-centered care and the logics of biomedical rationality and economic expediency. Our work can serve as a template for future research efforts focused on investigating person-centered care experiences of healthcare professionals in different care settings and services.

## Supporting information

S1 Appendix Interview guides. (DOCX)
