# Supplementary material for: Using a person-centered approach in clinical care for patients with complex chronic conditions: Perspectives from healthcare professionals caring for Veterans with COPD in the U.S. Veterans Health Administration’s Whole Health System of Care
Source: PLoS One. 2023 Jun 23;18(6):e0286326. doi: 10.1371/journal.pone.0286326 (PMC10289382; doi:10.1371/journal.pone.0286326)
Supplement: S1 Appendix — (DOCX) [file pone.0286326.s001.docx]

**APPENDIX 1**

# **Interview Guide for Clinicians**

**Background**

- What is your job/position title?
- What was your clinical training in?
- When did you complete your clinical training?
- How long have you worked in your current role?
- How long have you been with VA overall? Did you have other roles/positions?

**Personal practices with COPD care**

1. In what contexts/situations do you typically see Veterans with COPD? / How do Veterans with COPD wind up in your clinic?
2. Tell me about a typical clinical encounter with a Veteran with COPD. What kinds of things would you typically talk about with patients with COPD?
   1. How do you get the conversation started, typically?
3. What kinds of things are going through your mind during a typical encounter with a Veteran with COPD? What do you need to consider?
   1. What about the broader context of these Veterans’ lives (things that Veterans with COPD typically experience or live through)? Is this something that you consider? Can you give some examples?
4. [Time permitting] How do you see your role in addressing the needs of Veterans with COPD? What does your usual practice involve? / What sorts of things do you typically do? [Optional probes:
   1. By specialty (e.g., “How do you see your role as a primary care provider compared to a pulmonologist?”)
   2. By discipline (e.g., “How do you see your role as a NP compared to a physician?”)

**Whole Health**

*Now I want to shift fears a bit and ask you a few questions about Whole Health.*

1. First of all, have you heard of Whole Health? What does it mean to you?
   1. What is your opinion of Whole Health?
2. Have you had any training in Whole Health? Tell me about it.
3. Is there anyone in your clinic or in your broader social network who champions (goes above and beyond in promoting) Whole Health?
4. How, if at all, do you think a **Whole Health approach** may apply to COPD care? What might it look like?
   1. Do you see the way you provide care for Veterans with COPD as in line with the Whole Health approach? Why / why not?
5. As you may know, WH is about focusing on **what matters most** to patients. What do you think about that? Is this something that you do?
   1. **If yes** – How do you fit it into your clinical practice?
   2. **If no** – Could you tell me why not?
6. Are you familiar with personal health plans? Is this something you do in your practice – in general, or with Veterans with COPD?
   1. **[If yes]** Please tell me more about it.
      1. Why do you do this?
      2. How simple or complicated is it to do this? What kinds of things would make it easier of harder?
      3. How do you fit using the PHI into your clinical practice?
   2. **[If no – explain what it is and proceed with the following question and probes]** What do you think about this idea? Could you see yourself using it in future – why or why not?
      1. How simple or complicated would it be to do this?
      2. What could help you make it fit into your clinical practice?

**Care coordination and referrals**

*Now I want to ask you some questions about coordinating care for Veterans with COPD, including providing referrals.*

1. Which referrals do you typically provide to Veterans with COPD? Please walk me through your thought/decision process.
2. Thinking about these other specialties and services involved in managing patients with COPD, what is it like to coordinate care with them? What are some challenges? What works well?

*There are several specific services that I am particularly interested in.*

1. [Not to use with mental health care providers] The first of these services is mental health care. First of all, do Veterans ever bring up **mental health issues** or struggles with you? Is this something that you ask them about?
2. [Not to use with mental health care providers] Tell me about referrals to **mental health care**. Have you ever referred a Veteran with COPD to mental health care?
   1. [If yes:] Please tell me about it. Why do you refer Veterans to mental health care? What works well about it? What are some challenges, if any?
   2. [If not:] Why not? Can you see yourself providing a referral to mental health care in future? In which circumstances? If not, why not?
3. I want to ask about a different topic now. Has a patient ever brought up **spiritual or existential issues**? Is this something that you ask about?
4. Tell me about referrals to **chaplain services**. Have you ever referred a Veteran with COPD to see a chaplain?
   1. [If yes:] Please tell me about it. Why do you refer Veterans to chaplains? What works well about it? What are some challenges, if any?
   2. [If not:] Why not? Can you see yourself providing a referral to chaplain services in future? In which circumstances? If not, why not?
5. Tell me about referrals to **active mind-body classes**, such as yoga or tai chi. Have you referred a Veteran with COPD to such classes?
   1. [If yes:] Please tell me about it. Why do you refer Veterans to these classes? What works well about it? What are some challenges, if any?
   2. [If not:] Why not? Can you see yourself providing a referral to these classes in future? In which circumstances? If not, why not?
6. Thinking about the care that Veterans with COPD receive in outpatient settings at this VA as a whole, what do you think is done well? What is not done so well or could be improved? What are some gaps / “hiccups” in their care?
7. Is there anything else you’d like to tell me? Any last comments or things we didn’t cover?

# **Interview Guide for Whole Health Service Employees**

**Background**

- What is your job/position title?
- What was your training in?
- When did you complete your training?
- How long have you worked in your current role?
- How long have you been with VA overall? Did you have other roles/positions?

**Perspectives on WH clinical care**

1. What does a Whole Health approach in clinical care mean to you?
2. Thinking about Veterans with COPD in particular, why is it important that their clinical team use a WH approach? In what way is it helpful / What are the benefits to be derived?
3. Let’s imagine a situation in which a Veteran with a complex chronic condition, like COPD, comes to a VA primary care clinic for the first time. In the ideal world in which WH approach is fully implemented, what would that appointment look like?
   1. To the best of your knowledge, to what extent do the current practices in primary care clinics align with this ideal? What are the main challenges / barriers?
4. Let’s think about a similar situation: a Veteran with COPD comes to see a pulmonologist on a referral from primary care. What does that appointment look like if it is fully in line with WH clinical care ideal?
   1. To the best of your knowledge, to what extent do current practices in pulm clinics align with this ideal? What are the main challenges/ barriers?
5. We are hearing from providers that it may be difficult to have a “what matters most” conversation or to assess all areas of background. How would you respond?
6. What are your thoughts on where specialty care fits in with WH?
7. What is your perspective on the usefulness and feasibility of regular clinicians using a personal health plan?
8. Where does EHR fit in when it comes to WH clinical care?
9. How do you see the role of peers/coaches vs. clinicians? What is the ideal division of labor in team approach to WH?
10. Where does palliative care fit in relation to WH?

**Perspectives on CIH & referrals**

1. Where do CIH modalities come in when it comes to improving the quality of life of Veterans with complex chronic conditions, like COPD? / Again, thinking about the population of Veterans with COPD, what are the benefits they can derive from participating in CIH classes?
2. What are the current processes through which Veterans may sign up for CIH offerings? [Old, in house vs. new, contract-based approach] How well is that working?
3. What are some processes in place for providers to place referrals to CIH offerings, now that you have a new system?
4. Current barriers / challenges with that?
5. Where do you see chaplaincy fitting in?

For peer partners / coaches:

1. For this study, we are focusing on the population of Veterans with COPD. These folks typically suffer from breathlessness, chronic cough, and chronic fatigue as a result of chronic airflow obstruction that gets worse over time. It’s not unusual for them to have other chronic conditions, as well, including mental health issues. Have you ever worked with a Veteran who had COPD?
2. If yes: Can you tell me about a specific Veteran with COPD you’ve worked with? In what context was that? What concerns did he/she have? What goals did you set with him/her? Did the CIH modalities come up in the conversation? If so, tell me about it. If no, can you imagine yourself mentioning it in future? Did spiritual wellbeing/care come up? If so, tell me about it. If not, can you imagine yourself mentioning it in future?
3. If no: Tell me about an encounter with a Veteran who had a severe chronic illness, whose life was impacted by their illness a lot. [Continue with probes above]
4. How do you typically go about identifying Veterans’ concerns? How well does the PHI work for this process?
5. How do you typically go about setting or following up on goals? How well does the usual approach work?
6. How often do you see a PHP in Veterans’ record? How well do you think it works as it’s intended to, that is, as a living document that is accessible to the entire clinical team?
7. How often do you talk with Veterans about trying out CIH classes or appointments? Please tell me about this.
8. Do you ever refer Veterans to CIH classes/appointments? How well does that work? What could be improved?
9. How often do you talk with Veterans about spiritual wellbeing? How about seeing a chaplain? Ever put in a direct referral?

**Conclusion**

- What would you want to know about Veterans’ perspectives?
- Thinking about this VA as a whole, what is going well with integrating WH into clinical care? What are some “wins”?
- What are some barriers or persistent challenges?
- Who else on the team to speak to?
- Anything else to add?

# **Interview Guide for Chaplains**

**Background**

- What is your job/position title? [if unclear]
- How long have you worked in your current role?
- Can you tell me how you ended up becoming a VA chaplain?

1. What does your typical day look like?
2. Have you encountered Veterans with COPD in your work as a chaplain?
3. In your experience, what kind of **spiritual challenges** do Veterans with COPD face?
4. In what ways are chaplains **currently involved** in care for Veterans with COPD?
5. What about **outpatient settings** / outside of the hospital?
6. If your opinion, in what ways can spiritual care and chaplain services be **relevant** to supporting Veterans with COPD outside of the hospital?
7. To what extent, based on your experience, do clinicians at this VA currently take the time to **explore or assess** spiritual difficulties in Veterans with COPD or other life-limiting illnesses?
8. Is there currently a way for a provider to place a referral to a chaplain in an outpatient setting?
9. How do you see coordination between chaplaincy and other services, outside of medical services?
10. Anything else to add?
